# Effective Energy Efficiency under Delay–Outage Probability Constraints and F-Composite Fading

**DOI:** 10.3390/s24072328

**Published:** 2024-04-06

**Authors:** Fahad Qasmi, Irfan Muhammad, Hirley Alves, Matti Latva-aho

**Affiliations:** Centre for Wireless Communications, University of Oulu, 90014 Oulu, Finland; irfan.muhammad@oulu.fi (I.M.); hirley.alves@oulu.fi (H.A.); matti.latva-aho@oulu.fi (M.L.-a.)

**Keywords:** finite blocklength, effective capacity, radio resource management, MTC, EEE, QoS

## Abstract

The paradigm of the Next Generation cellular network (6G) and beyond is machine-type communications (MTCs), where numerous Internet of Things (IoT) devices operate autonomously without human intervention over wireless channels. IoT’s autonomous and energy-intensive characteristics highlight effective energy efficiency (EEE) as a crucial key performance indicator (KPI) of 6G. However, there is a lack of investigation on the EEE of random arrival traffic, which is the underlying platform for MTCs. In this work, we explore the distinct characteristics of F-composite fading channels, which specify the combined impact of multipath fading and shadowing. Furthermore, we evaluate the EEE over such fading under a finite blocklength regime and QoS constraints where IoT applications generate constant and sporadic traffic. We consider a point-to-point buffer-aided communication system model, where (1) an uplink transmission under a finite blocklength regime is examined; (2) we make realistic assumptions regarding the perfect channel state information (CSI) available at the receiver, and the channel is characterized by the F-composite fading model; and (3) due to its effectiveness and tractability, application data are found to have an average arrival rate calculated using Markovian sources models. To this end, we derive an exact closed-form expression for outage probability and the effective rate, which provides an accurate approximation for our analysis. Moreover, we determine the arrival and required service rates that satisfy the QoS constraints by applying effective bandwidth and capacity theories. The EEE is shown to be quasiconcave, with a trade-off between the transmit power and the rate for maximising the EEE. Measuring the impact of transmission power or rate individually is quite complex, but this complexity is further intensified when both variables are considered simultaneously. Thus, we formulate power allocation (PA) and rate allocation (RA) optimisation problems individually and jointly to maximise the EEE under a QoS constraint and solve such a problem numerically through a particle swarm optimization (PSO) algorithm. Finally, we examine the EEE performance in the context of line-of-sight and shadowing parameters.

## 1. Introduction

Massive machine-type communication (mMTC) and URLLC (ultrareliable low-latency communication) are the Next Generation cellular network use cases that promise to transform our societal landscape by introducing emerging applications and industry verticals. These two emerging communication use cases are mainly suited for the Internet of Things (IoT), thereby enabling their operation autonomously without any human interaction [1]. By 2025, over 50 billion devices will be interconnected through cellular access technologies [2]. The design of future wireless networks aims to achieve energy-efficient transmission while ensuring quality of service (QoS). Investigating and optimising the radio resources allocated during transmission is critical to maximise energy efficiency and throughput while guaranteeing reliability and latency.

To achieve the minimal delay requirements of most real-time verticals such as e-health, industrial IoT, and autonomous vehicles, a promising solution is to utilise short message communication. In this context, the lengths of the packets to be communicated are short, but their significance is high. With short packets under stringent QoS constraints, conventional performance metrics, such as the Shannon or outage capacity, offer a poor benchmark. Consequently, new and novel frameworks are required. In this context, the maximum achievable rate of finite blocklength packets was defined in [3] as a function of the blocklength and error probability. In [4], the authors recently explored covert millimetre-wave communications under a finite block length regime. They examined a scenario in which a transmitter, equipped with multiple antennas, sends covert messages to a legitimate receiver in the presence of spatially random wardens. Furthermore, the authors focused on identifying the optimal transmit power and block length to maximise the average effective covert throughput for beamforming strategies.

Furthermore, the traffic in the IoT ecosystem demands service guarantees in the time-varying wireless channel. The wireless channel is unpredictable due to environmental variations or obstacles [5], which can lead to significant violations of the QoS. Therefore, novel metrics are required to capture the model of the tail distribution of these traffic types. In this context, effective capacity (EC) and effective bandwidth (EB) are relevant metrics accounting for queuing, reliability, and (end-to-end) latency (unlike the Shannon capacity, which only considers the transmission rate). Therefore, EC is a powerful metric for low-latency communication that characterises the relation between the communication rate and the tail distribution of the packet delay violation probability [6]. Specifically, the effective capacity is defined as the highest arrival rate the network can serve under a particular delay constraint. On the other hand, the effective bandwidth characterises the minimum service rate required to support data arrival in a certain network subject to a QoS constraint [7].

To fully characterise the effect of the traffic on the wireless communication link, we need to look into three interconnected factors: message size, energy efficiency, and accurate environmental conditions. Concerning the first point, the IoT traffic comprises short and often burst messages, which are challenging to model using conventional wireless communication tools [8,9]. Therefore, short-packet transmission emerged in the past decade, thus extending the Shannon capacity with respect to the finite blocklength regime [10]. Regarding EC, Gursoy examined the statistical framework of the EC for a single node under a Rayleigh fading environment under a finite blocklength regime [11], thereby showing that the EC depends on the error probability and the delay QoS exponent. In [12], Musavian et al. maximised the EC in a cognitive radio network. Additionally, in [13], they examined the maximisation of the EC while considering the constraint of effective energy efficiency. In recent studies, in [14], the authors investigated the performance of the effective capacity under Markovian arrival traffic. They evaluated the system’s overall performance by considering the arrival and service processes. Later, the authors extended their analysis and assessed the performance under a finite blocklength regime while introducing an effective energy efficiency (EEE) model.

The second point is energy efficiency; in many applications, machine-type devices (MTDs) are battery-constrained and designed with strategies to maximise the network lifetime, thus impacting their behaviour and traffic [8,9]. For instance, an MTD may wake up, sense or sample, transmit its data in a burst, and then return to sleep mode. Such behavior affects the message size, the number of messages, the sensing/sampling capabilities, and the transmission strategies, thereby impacting the device activity and traffic. This is a cumbersome issue concerning activity detection and radio resource management for the base station (BS) [15]. In [16], the authors investigated secure energy efficient (SEE) beamforming in multibeam satellite (MS) systems, and they proposed a novel low-complexity optimisation framework, thereby aiming to maximise the SEE under the transmit power constraint. In focusing on energy efficiency, two energy-efficient models for cellular networks are attracting much interest in the literature. The first is based on the energy consumed per unit of network capacity, which is known as the energy efficiency model. It is a fundamental metric for evaluating the energy efficiency of a network [17]. This model characterises the maximum achievable throughput without considering the quality of service (QoS) requirements in IoT applications. Thus, it is mostly employed for assessing the efficiency of delay-insensitive services [18]. The second model is an effective energy efficiency model, which extends the concept of energy efficiency by incorporating the effective capacity, which measures the rate of reliable data transmission per unit of energy consumption under delay constraints. This formulation is accurate for supporting delay-sensitive IoT systems. In [13], the authors examined the energy efficiency problem in mMTC. They developed an analytical framework to measure energy efficiency, which was defined as the ratio of achievable rate to energy consumption. This measurement includes both radiator and static circuit powers. Shehab et al. [19] assessed the energy efficiency of delay-sensitive networks in the FBL regime and suggested an optimal power allocation technique.

The third point concerns the lack of accurate modelling of the device’s surrounding environment regarding multipath fading, shadowing, and their combined effect. The accurate modelling and analysis of fading channels are crucial for improving the reliability and efficiency of IoT communication networks. Recently, several distributions have been introduced, as they capture the intricate characteristics of the wireless communication medium beyond the multipath, as in Rayleigh or line-of-sight (LOS) components, as well as in Nakagami-*m* or Rician fading. For instance, the authors in [20] analysed the channel capacity in F-composite fading channels, thus introducing analytical models to calculate the capacity. The authors in [21] explored composite fading based on Rayleigh fading and inverse gamma shadowing, thereby offering theoretical insights and validation through practical data. Then, ref. [22] introduced an extended α−η−μ fading distribution and analysed the outage probability, average symbol error probability, effective rate, and average channel capacity. Likewise, ref. [23] assessed the second-order statistics of the Fisher–Snedecor distribution, notably in their application in burst error rate analysis of multihop communications, while [24] assessed the receiver switched diversity combining schemes under the same distribution. These studies are significant contributions with innovative models and analytical frameworks for understanding and mitigating fading effects in IoT networks, thus building reliable and efficient communication systems. However, they overlook latency-bounded metrics such as the EC.

### 1.1. Contribution

In [25], we employed the Nakagami-*m* fading model to examine the performance of EEE under a finite blocklength regime and QoS constraints, which specifically focused on the severity of the LOS. The simplicity of this model is advantageous for certain analyses, but it does not hold for practical scenarios where a strong LOS and some shadowing are present. As a result, our previous work may exhibit an optimistic outlook on EEE performance in practical conditions, where the combined influence of the LOS and shadowing significantly impacts signal propagation. This limitation highlights the need for more comprehensive models that can explicitly handle LOS and shadowing effects. Therefore, we consider the F-composite fading model, which enables us to understand how the performance of EEE is affected by the LOS and shadowing under QoS constraints. This model offers a more precise depiction of signal propagation. This progression in our research not only enhances the academic contribution to the field of wireless communications but also offers practical insights for designing and deploying EEE in environments where signal propagation conditions are less than ideal.

Our contribution addresses the three points by investigating the combined effects of multipath fading and shadowing on effective energy efficiency under a finite blocklength regime and QoS constraints, where IoT applications generate both constant and sporadic traffic. Our primary contributions are outlined below:

- We derive the exact outage probability expression for the F-composite fading channel under the FBL transmission regime, which is precise and tractable to capture the combined impact of multipath fading and shadowing.
- We confirm that the EEE is quasiconcave as a function of the signal-to-noise ratio (SNR).
- Our approach introduces power, rate, joint power, and rate allocation strategies that meet the stringent delay–outage constraints of random traffic while maximising the EEE.
- We provide numerical results that offer significant insight into the performance of cellular networks under different fading conditions. We notice a trade-off between the EEE and QoS constraints that is influenced by channel parameters such as the dominant signal component intensity and shadowing effect. This information will be crucial for those constructing future 6G network infrastructure to provide sufficient QoS for the IoT.
- We analyse the sporadic traffic arrivals at the transmitter under a nonempty buffer probability-based energy consumption model.
- Finally, the designed optimisation problem of maximizing the EEE is solved numerically through the PSO algorithm.

### 1.2. Outline

The remainder of this paper is organised as follows. In Section 2.1, we describe the network model, and we briefly review and define the F-composite fading channel model in Section 2.2. Section 2.3 summarises effective bandwidth and effective capacity. Later, in Section 2.4, we define the effective rate in a finite blocklength regime. Then, Section 2.5 introduces an MTC traffic model and analyses the average arrival rate. Section 3 introduces our novel analytical framework for outage probability, while Section 4 details energy efficiency for constant and bursty arrivals. The EEE maximisation problems and the different algorithms for obtaining solutions are discussed in Section 5. Then, selected numerical results appear in Section 6. Finally, Section 7 concludes the manuscript.

**Notation:** The expectation operator is denoted as $E\left[\cdot \right]$, while Γ(.) and Γ(.,.) are the gamma function [26] [Ch 6, 6.1.1] and the upper incomplete gamma function [26] [Ch 6, 6.5], respectively. B(a,b) and $2F_{1}\left(a,b;c;z\;\right)$ are the beta function [27] [8, 8.384.1] and the Gauss hypergeometric function  [27] [9, 9.111], respectively.

## 2. System Model

### 2.1. Network Model

**Figure 1:**
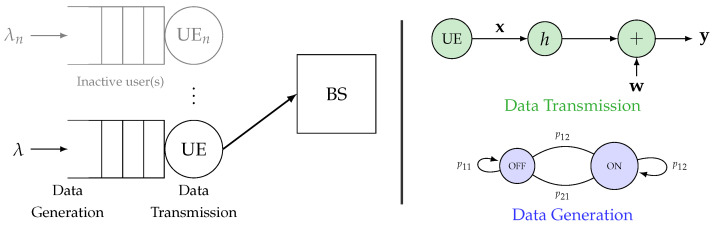
System Model: uplink of the MTC network, where the active user communicates with the BS. Note that λ is the arrival rate at the source’s transmission queue, and the data are generated according to a two-state discrete Markov process (indicated on the right). The transmit and received signals are x and y, respectively, while the channel coefficient is denoted as *h* and AWGN noise as w.

Assume an uplink-based MTC network comprising single-antenna energy-constrained IoT devices, usually known as user equipment (UE), sending seeing data to a central base station (BS). The sensor-generated data are stored in a buffer before transmission over a wireless channel, as shown in Figure 1, and the packets have small payloads. The received signal $y\in C^{n}$ comprises y=hx+w, where x is the transmitted signal, and w describes the complex, circularly symmetric, additive Gaussian noise with a unit mean and variance of $\sigma^{2}$. Moreover, note that h∈C denotes the quasistatic block-fading channel coefficient, i.e., it implies that h remains constant throughout the blocklength *n* but may vary from one block to another. Channel state information (CSI) is only available at the receiver. Thus, the transmitter sends the information at a fixed rate. Our goal in the manuscript is to establish a performance benchmark for the EEE. Noticed that we discuss the EEE framework for resource-constrained IoT devices. Our primary intuition is to evaluate the impact of traffic and combined effects of multipath fading and shadowing on the EEE model. This model might become more complex for the cost evaluation of the CSI at the receiver (CSIR). Therefore, it may masquerade certain impacts on the EEE. Hence, we consider the ideal case in which a perfect CSIR is available. Our obtained results under this notion provide upper bounds of the performance, and such an assumption allows us to focus on the impacts of traffic and different F-composite fading channel scenarios on the consumption models used in the EEE framework. However, the cost evaluation of the CSIR under the combined effects of multipath fading, shadowing, and a finite blocklength regime are interesting. It is a possible extension of our work, thus considering random access, channel estimation, and user detection in massive MTC scenarios.) of the system. However, evaluating the cost associated with CSI at the Receiver (CSIR) falls outside the scope of our current study.

### 2.2. Channel Model

With the broad spectrum of verticals envisioned for 6G communications, traditional cellular channel models, which usually account for only a single type of fading, are expected to be inadequate. In practice, a wireless channel undergoes multiple independent types of fading, which may or may not happen simultaneously. For instance, in downlink scenarios, the signal transmitted from the BS to the user equipment (UE) (i.e., MTD) undergoes two primary fading types. The first, known as large-scale fading or shadowing, is induced by large terrestrial objects, e.g., buildings or hills, thus causing unpredictable fluctuations in the overall signal strength. BSs are typically installed in elevated positions that are often clear of nearby obstructions. Conversely, UEs are usually located at lower elevations, and the LOS signal often encounters obstructions from nearby physical barriers, including the user’s own body. This results in a second type of fading known as small-scale fading that impacts the dominant signal component with random variations. Combining these two separate stochastic processes creates a very flexible composite fading channel model, which can effectively capture a wide range of shadowing and fading scenarios [28,29]. The F-composite fading model was recently proposed as an accurate and tractable statistical model for characterising composite fading conditions.

The network model described in Section 2.2 experiences a F-composite block-fading channel, where the received signal is generally interpreted as the product of two independent random processes, i.e., *Z* = *XY*, where *X* represents the Nakagami-*m* random variable with shape parameter *m*, and *Y* shows the normalized inverse Nakagami-*m* random variable with a $m_{s}$ shape parameter. The probability density function (PDF) and cumulative distribution function (CDF) of the instantaneous signal-to-noise ratio (SNR) γ in a F-composite fading channel are, respectively [20]

(1) fγ(γ)=mm(ms−1)msγ¯msγm−1Bm,msmγ+(ms−1)γ¯m+ms,Fγ(γ)=mm−1γmB(m,ms)(ms−1)mγ¯m F12m,m+ms,m+1;−mγ(ms−1)γ¯.

where $\bar{\gamma }$ represents the average SNR, and the instantaneous received SNR values γ[1],γ[2],… are treated as independent and identically distributed (IID) random variables (RVs) identical to an RV γ.

**Remark** **1.** *Regarding the physical interpretation of F-composite fading channel expressions, m signifies the multipath fading intensity. At the same time, $m_{s}$ is a shape parameter that governs the degree of shadowing experienced by the signal power. Lower values of m and $m_{s}$ imply deep fading and intense shadowing, respectively. As $m_{s}\to 0$, the scattered signal component experiences significant shadowing effects. On the other hand, as $m_{s}\to \infty$, the phenomenon of shadowing disappears from the wireless channel; thus, expressions converge to the standard Nakagami-m fading channel. Moreover, expressions depict Rayleigh fading specifically when m equals one and $m_{s}\to \infty$. Additionally, when both m and $m_{s}$ trend towards infinity, the F-composite fading model evolves towards a more deterministic behavior, i.e., it becomes equivalent to an additive white Gaussian noise (AWGN) channel. Finally, it is noted that these expressions remain valid when $m_{s}\geq 1$ and m≥0.5.*

### 2.3. Statistical Delay Constrained Analysis

We consider that the traffic produced by the random source is accumulated in a buffer before its transmission, which may cause a delay during the transmission due to data waiting in the buffer. Hence, the transmitter employs statistical QoS to control the buffer overflow probability [30].

(2) $$\begin{array}{c} \lim_{q\to \infty }\frac{\ln\;\mathrm{Pr}\{Q\geq q\}}{q}=-\theta , \end{array}$$

where *Q* represents stationary queue length of the buffer, *q* denotes the buffer over the threshold, and θ is the decay rate. The buffer overflow probability for relatively large *q* is approximated as

(3) $$\begin{array}{c} \mathrm{Pr}\left\{Q\geq q\right\}\approx P_{nb}\;e^{-\theta q}, \end{array}$$

where $P_{nb}=\mathrm{Pr}\left\{Q>0\right\}$ is the probability of a nonempty buffer. Notice that the probability of a nonempty buffer is often approximated by the ratio of the average arrival rate $\lambda_{\mathrm{avg}}$ to the average service rate [13]. The QoS exponent θ characterises the statistical QoS guarantees, and applications’ requirements always determine its value. A larger θ value corresponds to stringent constraints, and a smaller θ value shows looser constraints, which implies that the system can tolerate larger delays. Additionally, given the steady state queue delay *D* and *d* as a maximum tolerated delay, then the probability of delay violation is expressed as in  [13].

(4) $$\begin{array}{c} \mathrm{Pr}\left\{D\geq d\right\}\approx P_{nb}\;e^{-\theta a^{*}\left(\theta \right)d}, \end{array}$$

where $a^{*}\left(\theta \right)$ denotes the effective bandwidth [30].

### 2.4. Effective Rate at Finite Blocklength Regime

In this section, we explore the notion of the coding rate under the finite blocklength (FBL) regime. In a communication system, *k* bits of information are encoded into the *n* blocklength codeword with coding rate R=k/n. Subsequently, the codeword that has been formed by the encoder is transmitted via the wireless noisy (block fading) channel. Afterwards, the decoder at receiver side decodes the channel outputs and estimates of the information bits. As the codeword length approaches infinity, the communication becomes error-free at rates less than Shannon’s channel capacity. However, most of IoT applications generate short packets due to stringent latency constraints. Thus, the authors in [3] proposed an exact approximation that describes the probability of errors in a finite blocklength regime. The nodes, which generate short packets, utilise a novel achievable coding rate, denoted as *R*, while aiming to achieve a target error probability ϵ under *n* blocklength. The error probability is minimal but not zero. The normalized *R* in bits per channel (bpcu) is described as follows:

(5) $$\begin{array}{c} R\left(\bar{\gamma }\right)\approx \log_{2}(\;1+\bar{\gamma }{\left|h\right|}^{2}\;)-\frac{Q^{-1}\left(\epsilon \right)\log_{2}\left(\epsilon \right)}{\sqrt{n}}\sqrt{1-\frac{1}{(1+\bar{\gamma }{\left|h\right|}^{2}{)}^{2}}}, \end{array}$$

where $Q\left(.\right)=\int_{.}^{\infty }\frac{1}{\sqrt{2\pi }}e^{\frac{-t^{2}}{2}}dt$ represents the Gaussian Q function, $Q^{-1}$ signifies its inverse, and $\bar{\gamma }$ is the average signal-to-noise ratio. It is important to understand that the noise is normalized such that $\bar{\gamma }$ corresponds to the transmit power, and ${\left|h\right|}^{2}$ symbolises the squared envelope of the channel fading coefficients. The random variable $\gamma ={\left|h\right|}^{2}$ characterises the fading coefficients.

From (5), it is evident that the performance gap between Shannon’s channel capacity and the finite blocklength capacity narrows as *n* increases [3]. In practical scenarios, it is often assumed that the transmitter transmits information at a fixed rate due to its lack of knowledge of the channel coefficients. Alternatively, if the transmission rate varies for each fading block, it would introduce considerable complexity, which is why energy-limited IoT devices preferably transmit information at a fixed rate [31]. Hence, based on [32], for quasistatic fading channels, the outage probability is

(6) $$\begin{array}{c} \epsilon =E_{{\left|h\right|}^{2}}\left[Q\left(\frac{\log_{2}(1+\bar{\gamma }{\left|h\right|}^{2})-R}{\sqrt{\frac{1}{n}\left(1-\frac{1}{(1+\bar{\gamma }{\left|h\right|}^{2}{)}^{2}}\right)}\log_{2}e}\right)\right]. \end{array}$$

**Remark** **2.** *It is worth noting that the CSI at the transmitter (CSIT) may not be feasible for a massive MTC scenario, because the sensor is mostly uplink-oriented and uses sporadic transmissions such as those triggered by event-driven traffic, which typically have a low transmission rate. Because of these characteristics of the transmissions, devices avoid undergoing scheduling procedures, like grant-free random access [33]. Furthermore, such small devices are constrained by battery and baseband processing. Consequently, obtaining the CSIT would substantially drain energy resources [34]. Secondly, the receiver can acquire CSI from the dedicated training sequence and the codeword itself (combined estimation and decoding). Finally, even a minor variation in estimation at the transmitter can lead to significant interference and outage in the transmission. In contrast, a minor estimation error at the receiver adds a small noise term in the decoding procedure [31].*

It is assumed in [35] that the transmitter transmits data at a fixed rate. Given this assumption, the error probability ϵ varies based on the fading realisations. As a result, the data rate approaches zero in the presence of an outage; otherwise, it is nR. Given the above assumptions, the service rate (in bits per *n* channel uses) in each fading block is

(7) $$\begin{array}{c} R_{i}=\left\{\begin{array}{cc} 0 & \mathrm{with}\;\mathrm{prob}.\;\;\epsilon \\ nR & \mathrm{with}\;\mathrm{prob}.\;\;1\;-\epsilon . \end{array}\right. \end{array}$$

We can now articulate the effective rate, under a fixed rate and finite blocklength, as

(8) $$R_{E}\left(\theta ,n,R,\bar{\gamma }\right)=-\frac{1}{n\theta }\log_{e}\left\{\epsilon +\left(1-\epsilon \right)e^{-\theta nR}\right\}.$$

### 2.5. Traffic Model

The traffic arrival rate significantly impacts the EEE, system outages, and queuing delays [36]. Attaining an accurate approximation of these real-world arrival processes is challenging. Thus, we assume Markovian sources due to their effectiveness and tractability with respect to capturing constant and bursty arrival traffic, which are inherent to many IoT use cases [8]. We mainly consider a two-state ON-OFF model of discrete-time Markovian sources characterized by the time-discretized nature of data arrivals in the buffer [14].

In the ON state, λ bits arrive in the buffer, whereas in the OFF state, no data arrive, as shown in Figure 1. The system’s transition probabilities are defined by the matrix $J={\left(p\right)}_{ij}$, where $p_{11}\in \left[0;1\right]$ illustrates the probability of staying in the OFF state, while $p_{22}\in \left[0;1\right]$ determines the probability of the ON state. The transition probabilities between states are expressed as $p_{21}=1-p_{22}$ and $p_{12}=1-p_{11}$. In a steady state scenario, the probability of being in the ON state, denoted as $p_{on}$, is calculated as $p_{on}=\frac{1-p_{11}}{2-p_{11}-p_{22}}$ [30]. Consequently, the effective bandwidth for this two-state, discrete-time model is characterized as

(9) $$\begin{array}{cc} a^{*}\left(\theta \right) & =\frac{1}{\theta }\log_{e}\left(\frac{\left(\phi +\;\sqrt{\phi^{2}\;-\;4\left(p_{11}\;+\;p_{22}-1\right)e^{\lambda \theta }}\right)}{2}\;\right) \end{array}$$

(10) $$\begin{array}{cc} \; & \overset{\left(a\right)}{=}\frac{1}{\theta }\log_{e}\left(1-p+pe^{\lambda \theta }\right). \end{array}$$

In this model, ϕ is defined as $p_{11}+p_{22}e^{\lambda \theta }$, where (a) represents a simplified version of the effective bandwidth obtained by setting $p_{11}=1-p$ and $p_{22}=p$. This simplification results in the probability of the ON state $p_{on}$ being equal to *p* (refer to [30]), thereby introducing a single parameter, *p*, that serves as an indicator of the probability of data arrival. It accurately captures the variations in the arrival rate from one moment to another.

We intend to determine the average arrival rate of Markovian sources able to secure the effective rate while satisfying the QoS constraints (2). These QoS constraints are satisfied when the effective bandwidth of the arrival process is equal to the effective rate of the service process [30]. Hence,

(11) $$\begin{array}{c} a^{*}\left(\theta \right)=R_{E}\left(\theta ,n,R,\bar{\gamma }\right). \end{array}$$

To find the arrival rate that is capable of sustaining a transmission rate for specified values of *n*, $\bar{\gamma }$, and θ, we substitute the effective bandwidth formula of the discrete-time Markov source from Equation (9) into (11). We can derive the required arrival rate by substituting and solving for λ

(12) $$\begin{array}{c} \lambda =\frac{1}{\theta }\log_{e}\left(\frac{e^{\theta R_{E}\left(\theta ,n,R,\bar{\gamma }\right)}-\left(1-p\right)}{p}\right). \end{array}$$

Since the average arrival rate, denoted as $\lambda_{\mathrm{avg}}$, is the product of the arrival rate λ and the probability of being in the ON state $\lambda_{\mathrm{avg}}=\lambda \cdot p$, it equates to the average departure rate when the queue is in a steady state. Therefore, we can express the average arrival rate as a function of the quality of service (QoS) exponent θ, the effective rate, and the state transition probabilities [30]:

(13) $$\begin{array}{c} \lambda_{\mathrm{avg}}=\frac{p}{\theta }\log_{e}\left(\frac{e^{\theta R_{E}\left(\theta ,n,R,\bar{\gamma }\right)}-\left(1-p\right)}{p}\right). \end{array}$$

## 3. Closed-Form Expression of the Outage Probability

This section focuses on the outage probability of the channel model introduced in Section 2.2. The expectation of the outage probability expression in (6) is complex and cannot be easily obtained a closed-form expression, particularly when considering an SNR distribution as described in (1). Therefore, we obtained a tight approximation, which we will discuss next.

**Proposition** **1.** *Given the channel described in Section 2.2, the outage probability of the wireless link between the aggregator and IoT sensor is well approximated as*

(14) $$\epsilon_{ap}\;=\;\frac{F_{\gamma }\left(\vartheta \right)+F_{\gamma }\left(\varrho \right)}{2}+\;\frac{\psi \theta \left(F_{\gamma }\left(\vartheta \right)-F_{\gamma }\left(\varrho \right)\right)}{\sqrt{2\pi }}-\;\Psi ,$$

*where*

Ψ=ψmm(ms−1)γ¯ms−12π(1+m)B(m,ms)ϑm+1ϑm+κ2νF11,2−ms,2+m;ϑς−ϱm+1ϱm+κνF121,2−ms,2+m;ϱς,

*$\psi =\sqrt{\frac{n}{2\pi }}{\left(2^{2R}-1\right)}^{-\frac{1}{2}}$, $\alpha =2^{R}-1$, $\vartheta =\alpha +\sqrt{\frac{\pi }{2}\psi^{-2}}$, $\varrho =\alpha -\sqrt{\frac{\pi }{2}\psi^{-2}}$$\kappa =\left(m_{s}-1\right)\bar{\gamma }$, $\nu =1-m_{s}-m$, and $\varsigma =\frac{m}{\bar{\gamma }\left(1-m_{s}\right)}$.*

**Proof.**  The proof can be found in the Appendix A.    □

**Remark** **3.** *The expression in (14) is applicable across a diverse range of scenarios where the multipath fading and shadowing components play critical roles as exponents. It is important to highlight that (14) comprises only well-known functions, thus simplifying integrating through numerical methods compared to the original Equation (6).*

### On the Accuracy of (6)–(14)

**Figure 2:**
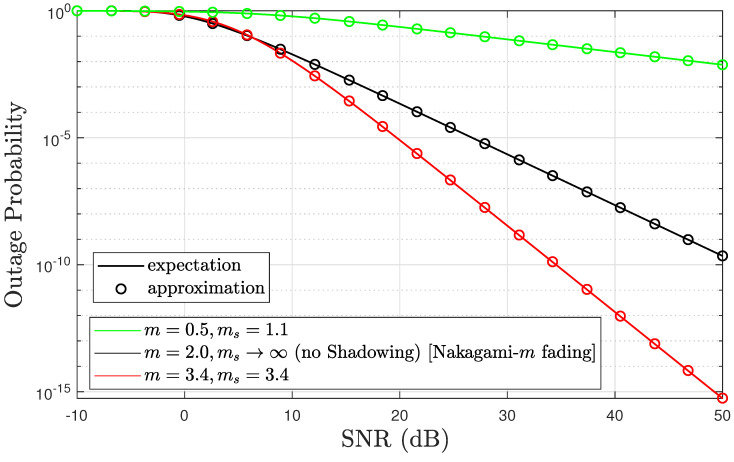
Comparing the accuracy of expectation and approximated outage probability expressions. This figure compares outage probability accuracy with the Q function’s numerical integral in (6) to the approximated outage probability in (14).

In this section, we add different F-composite fading channel scenarios to highlight better accuracy outcomes for the presented approximation, which are further evaluated against the numerical integrals of the Q function in (6), as illustrated in Figure 2. The results obtained from the closed-form approximate equations tightly match the numerical integral of the Q function in (6). Consider the following error metric:

(15) $$\Delta =|\frac{\epsilon -\epsilon_{ap}}{\epsilon }|.$$

Δ is almost always either zero or extremely near zero, which indicates the accuracy of the linearized Q function incorporated into the outage probability’s closed-form expression.

**Figure 3:**
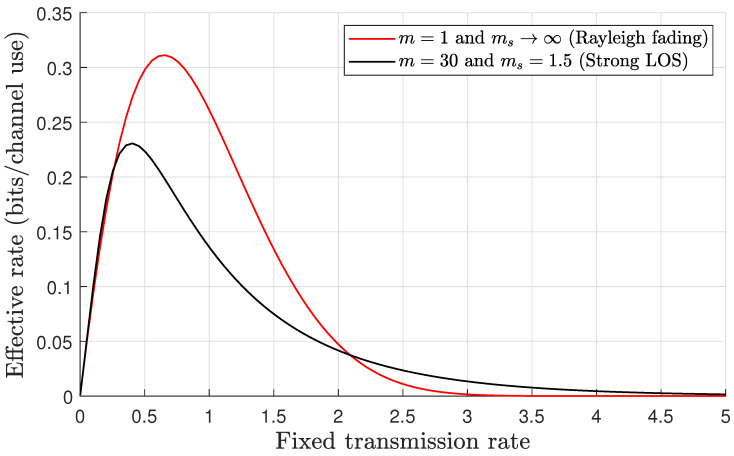
Effective rate vs. the fixed transmission rate *R* in the Rayleigh fading channel (when m=1 and $m_{s}\to \infty$, as in Figure 9 [35]), thus featuring strong LOS with strong shadowing. SNR=0 dB, θ=0.001, and the blocklength is n=1000.

**Remark** **4.** *It is evident that the results depicted in Figure 3 precisely match the results in Figure 9 [35] when m=1 and $m_{s}=\infty$ (Rayleigh fading channel). This match confirms our system model’s accuracy and reliability and highlights its capability to effectively capture the multipath fading with shadowing exponents. To further substantiate the clarity of our model’s capabilities, we have incorporated curves representing scenarios with a strong LOS and shadowing in which the effective rate curves exhibit quasiconcavity and are maximized at a unique value of R. These curve additions demonstrate the model’s robustness in capturing the combined impact of the LOS and shadowing (environmental conditions) on signal propagation.*

## 4. Effective Energy Efficiency

In our analysis, the energy efficiency of the system was measured in bits per joule, as recommended by ITU-R, and we utilised a linear power consumption model, which is characterised as [37]

(16) $$P_{t}=\xi \bar{\gamma }+P_{c},$$

where ξ represents the inverse drain efficiency of the transmission amplifier, and $P_{c}$ indicates the power dissipated by the hardware circuitry, which is measured in watts.

**Remark** **5.** *The linear power consumption model has been extensively employed in numerous research studies to analyse the energy efficiency in wireless systems [38]. This model accurately depicts the linear increase in power utilization due to the increase in transmit power and the inverse drain efficiency while also considering the power consumed when the circuit is idle. Moreover, this model supports our study objective of establishing a performance benchmark for the EEE in short-packet transmission under the QoS constraints of IoT devices. However, a limitation of this model is its assumption that the transmitter always has data for transmission.*

The performance benchmark for the EEE of the IoT is characterised as follows:

(17) $$\mathrm{EEE}=-\frac{1}{n\theta }\frac{\log_{e}\left\{\epsilon +\left(1-\epsilon \right)e^{-\theta nR}\right\}}{\xi \bar{\gamma }+P_{c}}=\frac{R_{E}\left(\theta ,n,R,\bar{\gamma }\right)}{P_{t}}.$$

In (16), the buffer seems to be always full while transmitting data. As a result, the transmitter is always in transmit mode, which leads to higher power consumption. This assumption is inaccurate and overestimates the energy efficiency. It is important to determine the transmitter’s mode while formulating the EEE for wireless fading channels under random data arrivals and statistical queuing constraints. Therefore, we explicitly considered two probabilistic events, namely the source’s arrival and the nonempty buffer probability, which cope with identifying the transmitter mode for transmission. As a result, we redefined the basic linear consumption model of (16) by incorporating the transmit mode with the transmission probability $p_{\mathrm{ptx}}$ and the idle mode with the idle probability $p_{\mathrm{idl}}$ [39].

(18) $$\begin{array}{cc} P_{t} & =\xi \bar{\gamma }\;p_{\mathrm{ptx}}+{\bar{\gamma }}_{\mathrm{idl}}p_{\mathrm{idl}}+P_{c}, \end{array}$$

By substituting $p_{\mathrm{ptx}}=1-p_{\mathrm{idl}}$ into Equation (18), we obtain the simplified power consumption model as follows:

(19) $$\begin{array}{c} P_{t}=\xi \bar{\gamma }-\left(\xi \bar{\gamma }-{\bar{\gamma }}_{\mathrm{idl}}\right)p_{\mathrm{idl}}+P_{c}. \end{array}$$

The probability of the transmitter being in an idle $p_{\mathrm{idl}}$ state depends on the probabilities of two events. The first event refers to when the source produces no traffic, and the second is when the buffer is empty.

(20) $$p_{\mathrm{idl}}=\left(1-p\right)\left(1-P_{nb}\right).$$

By incorporating $p_{\mathrm{idl}}$ in (19) with (20), we can reformulate the expression of the linear power consumption mode.

(21) $$P_{t}=\xi \bar{\gamma }-\left(\xi \bar{\gamma }-{\bar{\gamma }}_{\mathrm{idl}}\right)\left(1-p\right)\left(1-\frac{\lambda_{\mathrm{avg}}}{R}\right)+P_{c}.$$

It is observed that when $P_{nb}=1$ or p=1, the buffer is always full. Therefore, expressions (21) can be simplified to (16).

Therefore, the revised definition of the EEE is derived as

(22) $$\mathrm{EEE}=\;-\frac{1}{n\theta }\frac{\log_{e}\left\{\epsilon +\left(1-\epsilon \right)e^{-\theta nR}\right\}}{\xi \bar{\gamma }-\left(\xi \bar{\gamma }-{\bar{\gamma }}_{\mathrm{idl}}\right)\left(1-p\right)\left(1-\frac{\lambda_{\mathrm{avg}}}{R}\right)+P_{c}}.$$

### Model Validation

It is evident from [18,37] that the energy efficiency function rate will always be either non-negative or zero as the transmitting power approaches zero. Additionally, the function tends to zero as the transmitting power approaches infinity. This analysis further validates the correctness of the EEE, as expressed in (22), even in scenarios where the arrival traffic is sporadic.

**Lemma** **1.** *The upper bound of the EEE (22) is expressed as*

(23) $$\begin{array}{c} {\mathrm{EEE}}_{\infty }=\lim_{\bar{\gamma }\to \infty }\mathrm{EEE}=0, \end{array}$$

*which indicates that the EEE asymptotically approaches zero at a high SNR regime. Furthermore, at a low SNR, it is evident that the EEE converges to zero as follows:*

(24) $$\begin{array}{c} {\mathrm{EEE}}_{0}=\lim_{\bar{\gamma }\to 0}\mathrm{EEE}=0, \end{array}$$

**Proof.**  The proof can be found in the Appendix B.    □

**Figure 4:**
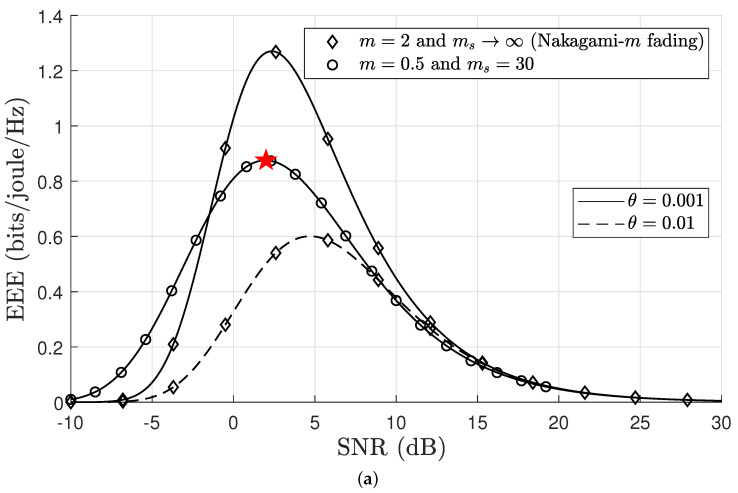

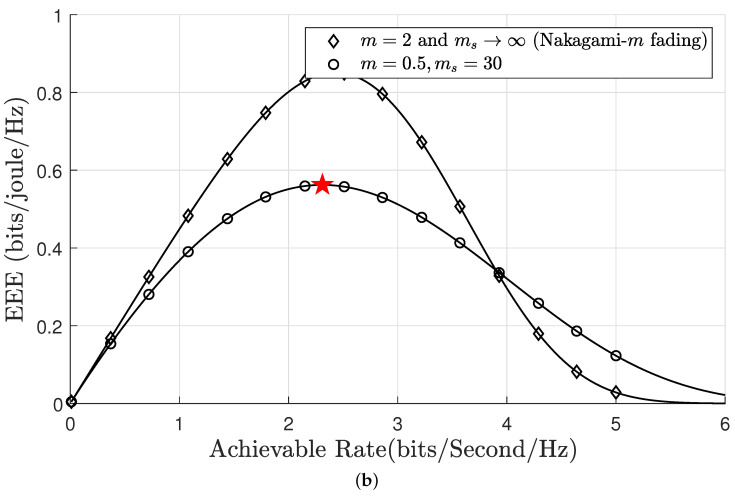
(**a**) EEE as a function of SNR for fixed vales of R=1, n=500, p=0.5, ξ=0.2, $P_{c}=0.2$ watts, and $p_{\mathrm{idl}}=0.03$ watts, where ★ denotes optimum EEE. (**b**) EEE as a function of rate for fixed vales of SNR=10 dB, n=500, p=0.5, θ=0.001, ξ=0.2, $P_{c}=0.2$ watts, and $p_{\mathrm{idl}}=0.03$ watts, where ★ denotes optimum EEE.

In the next analysis, by varying these variables, we investigated the impact of the transmission power $\bar{\gamma }$ and the rate *R* on the EEE. Figure 4a describes the effective energy efficiency (EEE) as a function of $\bar{\gamma }$, whereas Figure 4b expresses the EEE as a function of *R*, with fixed values of *m*, *n*, and varying the delay exponents θ. The results depicted in Figure 4a provide conclusive proof of an exact match with the findings presented in our earlier paper Figure 2 [25] under the conditions of m=2 and $m_{s}=\infty$ in a Nakagami-*m* fading channel. This exact correspondence verifies the correctness and dependability of our closed-form equation. It is noted that we are only focusing on one case in Figure 4, where $m=0.5,m_{s}=30$, and θ=0.001, in which a maximum point marks the optimum EEE as a ★, which is used as an exemplary example to facilitate our discussion of the results for obtaining the optimum EEE in Section 6. Furthermore, the analysis indicates that that EEE curves decline when a strict QoS constraint (θ→∞) is imposed on the buffer. The key intuition from Figure 4a,b reveals that the EEE exhibits quasiconcave characteristics with respect to both $\bar{\gamma }$ and *R*. More importantly, the evidence of quasiconcavity in the EEE is further substantiated by Lemma 1, which demonstrates that the EEE diminishes to zero with respect to $\bar{\gamma }$ [19]. The analysis confirms that the effective rate and power consumption are differentiable functions for *R* and $\bar{\gamma }$. Moreover, in the EEE expression, the denominator increases as $\bar{\gamma }$ increases and decreases as *R* decreases, which indicates the evident existence of quasiconcave characteristics. The effective usage of *R* and/or $\bar{\gamma }$ plays a crucial role in enhancing the energy efficiency of the communication system. The pursuit of the optimal pair $\left(R,\bar{\gamma }\right)$ is driven by improving the EEE, which will be further explored in the next section.

## 5. Maximisation of Effective Energy Efficiency with QoS Guarantees

In power-limited systems, optimising radio resources is critical for ensuring QoS guarantees and energy saving simultaneously. The transmission power and rate radio resources generally form sophisticated radio management schemes. It is important to note that hardware limitations constrain power allocation, as the transmission hardware must operate with upper and lower power limits. Additionally, the transmission rate offers an opportunity for optimisation, especially in scenarios where devices transmit data at varying rates. This flexibility in the transmission rate offers a valuable degree of freedom (DoF) for optimisation. Such resource allocation strategies have been implemented in different configurations. For instance, LPWAN (low-power wide area network) INGENU technologies often use power allocation to extend battery life [40]. Conversely, LoRa typically employs rate control strategies coupled with fixed transmission power levels [41,42].

We have presented various formulations of the EEE maximisation problem to identify optimal resource allocation by exploiting the transmission power and rate radio resources. These formulations involve different strategies, including minimal transmission power allocation (PA), maximal rate allocation (RA), or a combination of PA and RA. Additionally, it is important to note that the transmission power is constrained on the upper bound denoted by $P_{max}$.

Hence, the optimisation problems can be formulated as P1, P2, and P3. Initially, we formulated the power allocation (PA) problem as P1, where the primary objective was to determine the optimal power ${\bar{\gamma }}^{*}$ that satisfies the QoS constraints and simultaneously yields the maximum EEE for a fixed transmission rate *R*.

(25) $$\begin{array}{cc} P1:\;{\mathrm{maximize}}_{\bar{\gamma }\in S}\; & \mathrm{EEE}, \end{array}$$

(26) $$\begin{array}{cc} \mathrm{subject}\;\mathrm{to}\; & \bar{\gamma }\leq P_{max}. \end{array}$$

where $S\subseteq R_{+}$, $\mathrm{EEE},R_{E}\left(\theta ,n,R,\bar{\gamma }\right):S\to R_{+},\;\mathrm{EEE}\geq 0,\;R_{E}\left(\theta ,n,R,\bar{\gamma }\right)\geq 0$. We then reformulated the rate allocation (RA) problem as P2, where the goal was to identify the optimum rate, ${\bar{\gamma }}^{*}$ and maximise the EEE for a given fixed power level $\bar{\gamma }$ while also guaranteeing the QoS constraints.

(27) $$\begin{array}{cc} P2:\;{\mathrm{maximize}}_{R\in S}\; & \mathrm{EEE}, \end{array}$$

(28) $$\begin{array}{cc} \mathrm{subject}\;\mathrm{to}\; & \bar{\gamma }\leq P_{max}. \end{array}$$

Lastly, we introduced the problem P3, which simultaneously identifies transmission powers and rates (PA and RA). This approach is designed to maximise the EEE under QoS constraints. P3 is informed by the insights and results obtained from the previously addressed problems: P1 and P2.

(29) $$\begin{array}{cc} P3:\;{\mathrm{maximize}}_{\bar{\gamma }\in S,R\in S}\; & \mathrm{EEE}, \end{array}$$

(30) $$\begin{array}{cc} \mathrm{subject}\;\mathrm{to}\; & \bar{\gamma }\leq P_{max}. \end{array}$$

The complexity of the EEE expression makes the optimisation problem particularly challenging, especially when obtaining a closed-form solution. It is important to note that problems P1, P2, and P3 each comprise a ratio of two functions, the effective rate $R_{E}$ and the power consumption $P_{t}$, both of which are dependent on *R* and $\bar{\gamma }$.

### Particle Swarm Optimisation

Particle swarm optimisation (PSO) is a nature-inspired swarm intelligence-based algorithm (Algorithm 1) that mimics the collective behaviour of birds flocking to explore and exploit the multidimensional search space for food. This algorithm employs the principles of self-organisation exhibited by the number of agents called particles. Every particle is characterised by a specific position and velocity within the search space, which are instrumental in calculating potential solutions for the optimisation problem. This algorithm promises to find the global optimum of an objective function by iteratively adjusting the positions and velocities of the agents inside the problem domain. A fitness function is an objective function that is needed to grade the quality of a solution by assessing each particle that yields the most optimal evaluation of the given fitness function [43]. Let us define the objective function as

$$\begin{array}{cc} f_{\mathrm{PSO}}\left(\varphi \right)=\;{\mathrm{maximize}}_{\varphi \in S}\; & \mathrm{EEE}, \end{array}$$

During the initialisation phase, every particle is assigned a random position φ along with a velocity ∅ that enables it to traverse inside the search space. In each iteration, every particle independently calculates its personal best pBest, as well as the global best solution, known as gBest. To converge on the optimal global best solution, the algorithm employs a dual strategy that incorporates both pBest and gBest in the following equations for iteratively adjusting the velocity and position of each particle [44].

(31) $$\begin{array}{c} \chi =\iota \chi +C_{1}\tau_{1}\left(\mathrm{pBest}-\varphi \right)+C_{2}\tau_{2}\left(\mathrm{gBest}-\varphi \right) \end{array}$$

(32) $$\begin{array}{c} \varphi =\varphi +\chi \end{array}$$

The variable ι represents the inertia weight, which is between 0 and 1. The acceleration coefficients are $C_{1}$ and $C_{2}$, where $C_{1}$ and $C_{2}$ are both between 0 and 2 and inclusive, and $\tau_{1}$ and $\tau_{2}$ are the randomly generated values. The updating method is iterated until it converges to a desirable gBest value. Upon receiving the new updated position, the particle assesses the fitness function and subsequently updates pBest and gBest for the minimisation issue in the following manner.**Algorithm 1** Particle Swarm Optimisation (PSO) Algorithm1:Initialisationparameters:  maximum number of iterations $\max_{n}$ι, $C_{1}$, $C_{2}$, $x_{max}$, $x_{min}$2:Initialisationparticlesandvelocities:  φ←Randomly initialise number of particles within [$x_{max}$, $x_{min}$]  χ← Randomly initialise number of particles velocities3:Initialisationpersonalbestandglobalbest:  pBest ←φ  pBestValue $\leftarrow f_{\mathrm{PSO}}$(pBest)  [gBestValue, gBestIndex] ← Max(pBestValue)  gBest ←φ[gBestIndex]  FOR i from 1 to $\max_{n}$ DO4:Updatevelocities:  χ←ιχ + $C_{1}$$\tau_{1}$(pBest −φ) + $C_{2}$$\tau_{2}$ (gBest −φ)5:Updateparticlepositions:  φ←φ + χ6:BoundaryConditions:  φ← Clip(φ, $x_{max}$, $x_{min}$)7:EvaluateObjectiveFunction:  δ←$f_{\mathrm{PSO}}$(φ)8:UpdatePersonalBest:  updateIndices ←δ > pBestValue  pBest[updateIndices] ←φ[updateIndices]  pBestValue[updateIndices] ←δ[updateIndices]9:UpdateGlobalBest:  (nGBestValue,nGBestIndex) ← Max(pBestValue)  IF nGBestValue > gBestValue THEN  gBestValue ← nGBestValue  gBest ← particles[nGBestIndex]  END IF  END FOR

## 6. Results and Discussion

This section presents the numerical results of the optimal resource allocation strategies, and we assessed their performance across five different F-composite fading channel scenarios. These scenarios are as follows: (i). In a heavy shadowing scenario $\left(m=30,m_{s}=1.1\right)$, a transmitter and receiver are in an urban setting characterised by high-rise buildings and dense greenery. Despite their proximity and a clear line of sight, the signal path experiences substantial shadowing due to obstructions. (ii). For severe multipath fading $\left(m=0.5,m_{s}=30\right)$, the transmitter and receiver are situated in an open field where no significant obstructions interfere with their line of sight. However, the line of sight between the two devices is weak due to distance or terrain variations. (iii). An intense composite fading scenario $\left(m=0.5,m_{s}=1.1\right)$ featuring both heavy shadowing and no LOS, which can be commonly observed in indoor environments, such as within large office buildings, shopping malls, or underground facilities like subway stations. (iv). A scenario of light composite fading $\left(m=50,m_{s}=50\right)$, where there is an LOS and no shadowing typically occurs in flat, open areas with minimal physical obstructions, such as deserts, plains, or certain coastal regions. (v). A moderate composite fading scenario $\left(m=3.4,m_{s}=3.4\right)$, commonly encountered in suburban areas or semiurban environments. Open spaces and physical obstructions, such as small homes, buildings, trees, and terrain, characterise this setting.

Initially, we examined the effects of LOS and shadowing conditions on the EEE. It is also noted that a constant data arrival rate increases the $\lambda_{\mathrm{avg}}$, thus leading to higher energy consumption than a lower arrival rate. Finally, we explored the trade-off between EEE and the probability of outage delay violations. Regarding the simulation results, we set the network parameters as follows: $P_{c}$ and $p_{\mathrm{idl}}$ were set to 0.2 and 0.03 watts, ξ=0.2, d=500, and n=500. While solving P1 and P2, we assumed R=1 bps/Hz and $\bar{\gamma }=10$ dB respectively.

**Figure 5:**
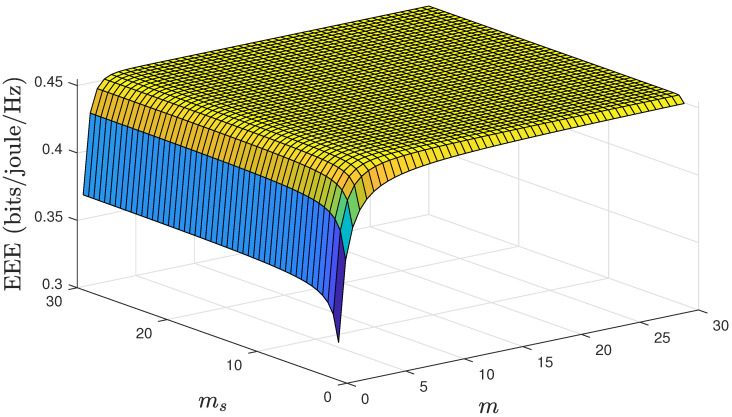
EEE as a function of the *m* and $m_{s}$ for fixed values of n=500, d=500, and θ=0.001.

Next, as shown in Figure 5, we investigated the impact of LOS and shadowing characteristics of the F-composite fading channel on the EEE. The EEE seems to be more sensitive to LOS than shadowing. Therefore, while designing the system, it is recommended to consider factors such as antenna location and height or to use technology that improves the direct LOS. Furthermore, it is noticed that after a certain point, further improvements in the LOS conditions do not translate into proportional gains in the EEE; this is attributed to QoS constraints, which impose an upper bound on the achievable effective rate, as well as on the EEE.

### 6.1. Optimal EEE under Markovian Arrivals

**Figure 6:**
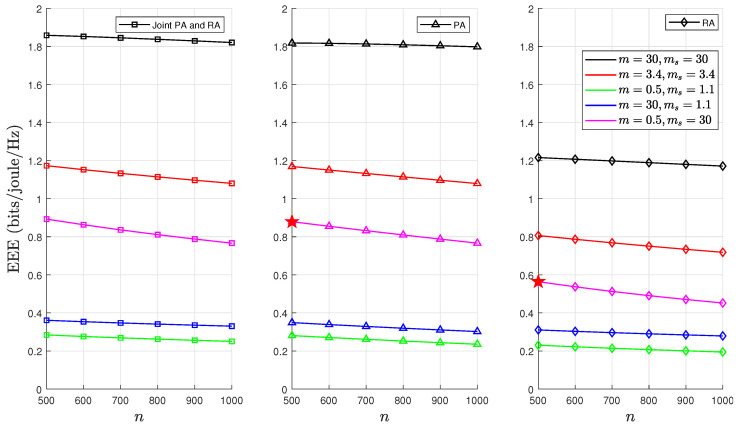
EEE as a function of *n* for fixed values of d=500, p=0.5, and θ=0.001, where ★ denotes optimum EEE.

**Figure 7:**
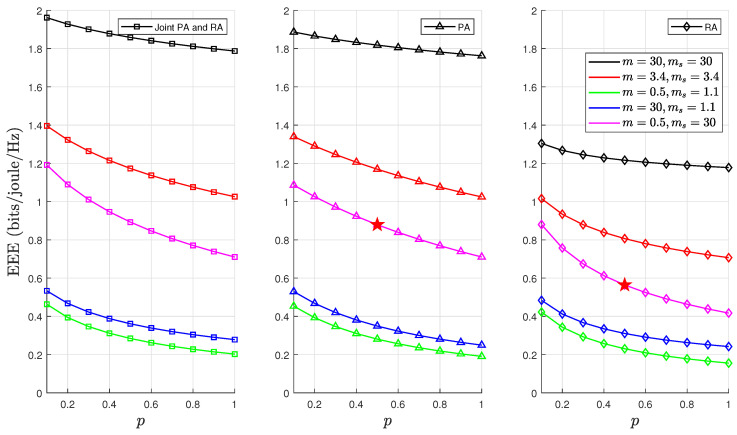
EEE as a function of *p* for fixed values of d=500, n=500, and θ=0.001, where ★ denotes optimum EEE.

**Figure 8:**
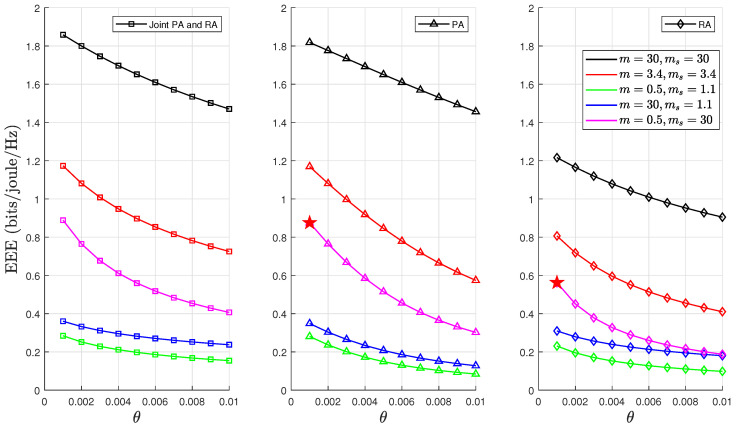
EEE as a function of θ for fixed values of n=500, d=500, and p=0.5, where ★ denotes optimum EEE.

We highlight that the results in Section 6.1 present optimal EEE values for PA, RA, and joint PA and RA schemes under five different F-composite fading channel scenarios. These results were obtained through the PSO algorithm detailed in Algorithm 1. A comprehensive and clear analysis of Figure 4, Figure 6, Figure 7 and Figure 8 is required to substantiate the optimality of the resource allocation strategies proposed by the PSO algorithm. In Figure 4, the optimal EEE values are depicted. We focused on one case, where $m=0.5,m_{s}=30,\theta =0.001$, and p=0.5, in which a maximum point marked optimum EEE as ★ has been used as an exemplary example to facilitate our discussion. This explicit marking of ’optimum EEE’ values ensures visibility for an unambiguous understanding. The validation continues with Figure 6, Figure 7 and Figure 8, which further corroborate these results by demonstrating the optimal EEE values derived via the PSO algorithm converged closest to the optimal EEE value, which is marked in Figure 4. The uniformity of the results across these figures not only illustrates the accuracy of the PSO algorithm but also confirms the optimality of different resource allocation strategic.

Figure 6 demonstrates how the performance of the optimal EEE varies as a function of the blocklength *n* parameter over the F-composite fading channels with five different combinations of the *m* and ms parameters. It has been noted that a lower value of *n* improved the EEE due to the lower allocation of channel resources for transmitting packets. In light composite fading scenarios, the probability of buffer congestion decreases significantly. This occurs because as the values of the *m* and ms parameters increase, the composite fading channel becomes more identical to an AWGN channel. This leads to a rise in the EEE, particularly when compared with intense composite fading. Conversely, the EEE was adversely affected when the channel was subjected to severe multipath fading and shadowing, i.e., intense composite fading. Interestingly, the EEE was higher in severe multipath fading scenarios than in heavy shadowing. This indicates that shadowing has more detrimental effects on the performance of mMTC, especially when QoS constraints are imposed on the buffer. Moreover, it was determined that the joint PA and RA achieved better performance compared to the individual PA or RA, particularly when there were relaxed QoS constraints. This is because under loose QoS constraints, the joint PA and RA strategy has more degrees of freedom in the optimal selection of the rate and power. Moreover, it has been determined that combined rate and power optimisation outperformed each strategy alone or the power optimisation.

Figure 7 demonstrates how the optimal EEE varies with the parameter *p* under fixed values of the QoS constraint θ and constant *n*. The variations in the average value of $\lambda_{\mathrm{avg}}$ were directly controlled by the parameter *p*. Consequently, it was observed that *p* captured both consistent and irregular traffic patterns that are characteristic of IoT devices. Consequently, different $\lambda_{\mathrm{avg}}$ variations led to distinct optimal power and rate solutions in the optimisation problems P1, P2, and P3. An analysis of the impact of $\lambda_{\mathrm{avg}}$ on the EEE reveals that higher traffic arrival rates led to increased energy consumption compared to lower rates due to more packets accumulating in the buffer for transmission. Moreover, a combined PA and RA strategy was more effective than other methods in handling sporadic traffic. The probability of buffer congestion was reduced in scenarios with light composite fading. This is primarily because the F-composite fading channel becomes more deterministic, which consequently delays violation and outage probability.

Figure 8 demonstrates how the optimum EEE varies as a function of the delay constraint θ over the F-composite fading channels. The value of θ affected the EEE considerably, with the impact on intense fading conditions being the most detrimental. The EEE decreased with stringent QoS constraints in all considered fading conditions because looser delay constraints reduce the possibility of buffer congestion, thereby leading to a higher EEE than the stringent QoS constraints. As expected, for both the joint PA and RA and PA strategies, better performance was achieved across all fading conditions.

**Figure 9:**
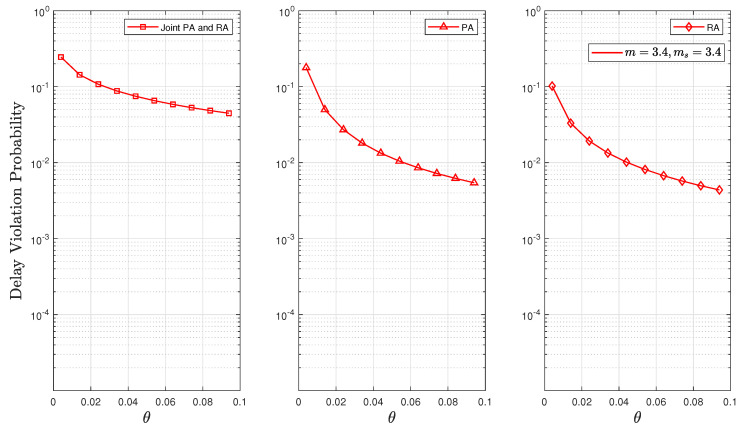
Delay violation probability as a function of θ for fixed values of n=500, d=500, and p=0.1.

**Figure 10:**
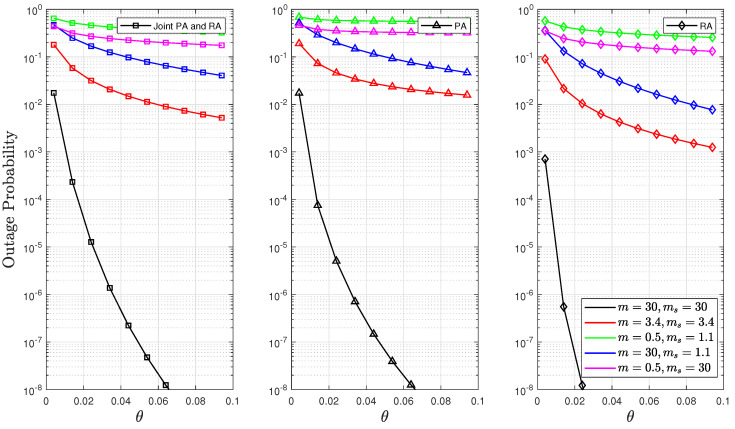
Outage probability as a function of θ for fixed values of d=500, n=500, and p=0.1.

Figure 9 and Figure 10 investigate the effect of the QoS exponent θ on outage and delay violation probabilities. These figures depict the solutions to the optimisation problems P1, P2, and P3 for θ. These results demonstrate that employing either a PA or RA strategy resulted in lower probabilities of delay and outage as the value of θ increased. This is because of our assumption regarding the fixed value of the SNR of (i.e., $\bar{\gamma }=10$ dB) in the RA strategy, which led to the higher values of the optimal rates of *R* and the effective rates compared to the strategy of joint PA and RA. Therefore, these higher values significantly decreased the delay violation probability (given that the delay violation probability function is inversely proportional to *R*, as defined in (4)). The findings substantiate this observation in Lemma 1. Additionally, it was observed that the possibility of buffer congestion was reduced under light composite fading scenarios, thus decreasing both the delay violation and outage probabilities. This effect is attributed to the channel becoming more deterministic in such scenarios.

**Figure 11:**
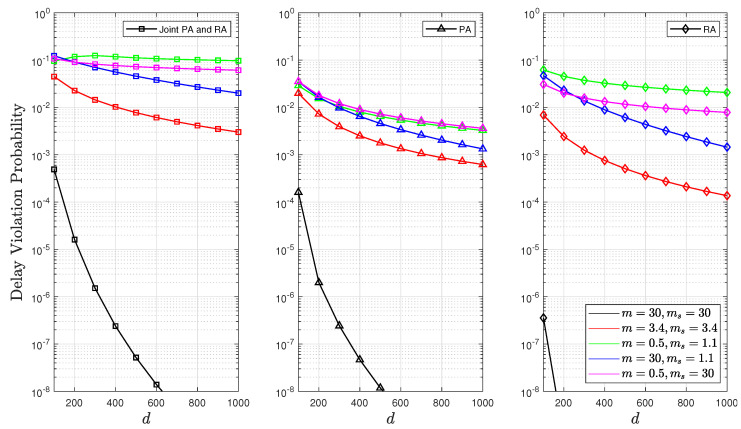
Delay violation probability as a function of *d* for fixed values of n=500, θ=0.1, and p=0.1.

Figure 11 depicts how the probability of delay violation varies with different delay bounds *d*. A larger value of *d* indicates that the system can tolerate longer delays, thus decreasing the probability of delay violation as *d* increases. Additionally, it was evident that intense composite fading consumed more energy and had lowered effective rates and increased packet buffering time due to slower transmission rates (buffer congestion), which subsequently increased the probability of the nonempty buffer $P_{nb}$. This may result in a higher delay violation probability function. This finding aligns with the observations in Figure 9 showing that a joint PA and RA optimisation strategy resulted in a higher probability of delay violation. In contrast, using RA alone reduced the probability of delay violation. This is attributed to the high fixed value of $\bar{\gamma }$ in RA, which is a conclusion also supported by Lemma 1.

## 7. Conclusions

In this work, we presented a comprehensive EEE analysis via F-composite fading. In this context, we derived an accurate approximation of outage probability, and our simulation results validate the correctness of our expression. We designed an energy-efficient power and rate allocation scheme for a point-to-point buffer-aided communication system model under QoS constraints based on the EEE expression. The numerical results disclose that slight variations in the QoS constraints, the buffer conditions, and the degree of multipath fading and shadowing significantly impact the EEE. The study also examined the impact of various composite fading scenarios on the EEE. Moreover, it seems that the EEE is more sensitive to the LOS than to shadowing. Therefore, when designing the system, it is recommended to consider factors such as antenna location and height or to use technology that improves the direct LOS. Our results provide a crucial understanding of how traffic impacts the efficiency of communication networks. We demonstrated that a joint PA and RA constitutes an optimal resource allocation strategy. However, this strategy may result in higher outage and delay violation probabilities because of fixed values of assumption for the SNR (i.e., $\bar{\gamma }=10$ dB) in the RA and 1 bps/Hz in PA. Finally, it should be emphasized that the proposed EEE framework introduced in this study has practical applications for 6G communication scenarios. These applications are particularly relevant where traditional Rayleigh or Nakagami-*m* fading models cannot capture the channel’s diverse characteristics. 

## Data Availability

Data are contained within the article.

## Abbreviations

The following abbreviations are used in this manuscript:

**Symbol**	**Definition**
EEE	effective energy efficiency
NBP	nonempty buffer probability
FBP	full buffer probability
FBL	finite blocklength
*h*	fading coefficient
*D*	queueing delay
*d*	maximum delay
$P_{nb}$	nonempty buffer probability
θ	delay exponent
$a^{*}$	effective bandwidth
$R_{E}$	effective capacity/effective rate
*m*	fading parameter
$m_{s}$	shadowing parameter
ϵ	outage probability
$\bar{\gamma }$	average signal-to-noise ratio
γ	instantaneous signal-to-noise ratio
*R*	achievable channel rate
*n*	blocklength
λ	arrival rate
$\lambda_{\mathrm{avg}}$	average arrival rate
*p*	arrival probability
ξ	inverse drain efficiency
$P_{c}$	hardware power dissipated
PA	power allocation
RA	rate allocation

## Appendix A. Proof of Proposition 1

According to [32], it is possible to tightly approximate the function Q(g(t)) with a linear function across the entire SNR range. It is important to note that the argument within the Q function, as specified in (6), is provided as $g\left(t\right)=\sqrt{n}{\left(1-{\left(1+t\right)}^{-2}\right)}^{-\frac{1}{2}}\log\left(1+t\right)$, and it is observed that g(t) increases for *t*, but it is not strictly positive ∀t∈R, which restricts the use of other well-known approximations for the Q function [32].

Then, let Q(g(t))≈W(t) be referred to as

(A1) $$\begin{array}{c} W\left(t\right)=\left\{\begin{array}{cc} 1 & t\leq \varrho \\ \frac{1}{2}-\frac{\psi }{\sqrt{2\pi }}\left(t-\alpha \right) & \varrho <t<\vartheta \\ 0 & t\geq \vartheta \end{array}\right. \end{array}$$

where $\theta =2^{R}-1$ is the solution of g(t)=0, while $\psi =\sqrt{\frac{n}{2\pi }}{\left(2^{2R}-1\right)}^{-\frac{1}{2}}$ is the solution for $\frac{\partial Q(g(t))}{\partial t}{|}_{t=\theta }$.

Subsequently, the outage probability is given by

$$\begin{array}{cc} \epsilon & =E_{\gamma }\left[\epsilon \right]=\int_{0}^{\infty }W\left(\gamma \right)f_{\gamma }\left(\gamma \right)dz\\ \; & =\int_{0}^{\varrho }\left(f_{\gamma }\left(\varrho \right)\right)d\gamma +\int_{\varrho }^{\vartheta }\left(\frac{1}{2}-\frac{\psi }{\sqrt{2\pi }}\left(\gamma -\alpha \right)\right)f_{\gamma }\left(\gamma \right)dz\\ \; & =F_{\gamma }\left(\gamma \right){|}_{0}^{\varrho }+\int_{\varrho }^{\vartheta }\left(\frac{1}{2}-\frac{\psi }{\sqrt{2\pi }}\left(\gamma -\alpha \right)\right)f_{\gamma }\left(\gamma \right)d\gamma \\ \; & =F_{\gamma }\left(\varrho \right)+\frac{1}{2}F_{\gamma }\left(\gamma \right){|}_{\vartheta }^{\varrho }+\frac{\psi \alpha }{\sqrt{2\pi }}F_{\gamma }\left(\gamma \right){|}_{\vartheta }^{\varrho }-\int_{\varrho }^{\vartheta }\frac{\psi \gamma }{\sqrt{2\pi }}f_{\gamma }\left(\gamma \right)d\gamma ,\\ \; & =\frac{F_{\gamma }\left(\vartheta \right)+F_{\gamma }\left(\varrho \right)}{2}+\frac{\psi \alpha }{\sqrt{2\pi }}\left\{F_{\gamma }\left(\vartheta \right)-F_{\gamma }\left(\varrho \right)\right\}-I. \end{array}$$

Notice that *I* can be expressed as

(A2) $$\begin{array}{cc} I & =\frac{\psi }{\sqrt{2\pi }}\int_{\varrho }^{\vartheta }\gamma f_{\gamma }\left(\gamma \right)d\gamma \\ \; & =\frac{\psi }{\sqrt{2\pi }}\frac{m^{m}{\left(m_{s}-1\right)}^{-m}{\bar{\gamma }}^{-m}}{B(m,m_{s})}\int_{\varrho }^{\vartheta }\frac{\gamma^{m}}{{\left[\frac{m\gamma }{\left(m_{s}-1\right)\bar{\gamma }}+1\right]}^{m+m_{s}}}d\gamma . \end{array}$$

Note that (A2) is obtained after a few algebraic manipulations; once the integral is in this form, we resort to (3194.1) [27] and perform parameter simplification in the F12 hypergeometric function [45] to obtain the closed form approximation as shown in (14).

## Appendix B. Proof of Lemma 1

From (14) and Figure 2, the outage probability bound can be exhibited as $\lim_{\bar{\gamma }\to 0}\epsilon =1$ and $\lim_{\bar{\gamma }\to \infty }\epsilon =0$. Then,

$$\begin{array}{cc} \lim_{\bar{\gamma }\to 0}C_{E}\left(\theta ,n,R,\bar{\gamma }\right) & =-\frac{1}{n\theta }\log_{e}\left\{\epsilon +\left(1-\epsilon \right)e^{-\theta nR}\right\}=0.\\ \lim_{\bar{\gamma }\to \infty }C_{E}\left(\theta ,n,R,\bar{\gamma }\right) & =-\frac{1}{n\theta }\log_{e}\left\{0+\left(1-0\right)e^{-\theta nR}\right\}=R. \end{array}$$

Based on the above, the EEE (at zero ${\mathrm{EEE}}_{0}$ and at infinity ${\mathrm{EEE}}_{\infty }$) becomes

$$\begin{array}{cc} {\mathrm{EEE}}_{0} & =\lim_{\bar{\gamma }\to 0}\mathrm{EEE}=0.\\ {\mathrm{EEE}}_{\infty } & =\lim_{\bar{\gamma }\to \infty }\mathrm{EEE}=0. \end{array}$$

which concludes the proof.
